# Treatment Adherence of Tuberculosis Patients Attending Two Reference Units in Equatorial Guinea

**DOI:** 10.1371/journal.pone.0161995

**Published:** 2016-09-13

**Authors:** Gabriela Fagundez, Hugo Perez-Freixo, Juan Eyene, Juan Carlos Momo, Lucia Biyé, Teodoro Esono, Marcial Ondó Mba Ayecab, Agustín Benito, Pilar Aparicio, Zaida Herrador

**Affiliations:** 1 Department of Preventive Medicine, Hospital Universitario 12 de Octubre, Madrid, Spain; 2 Department of Preventive Medicine, Hospital Clínico Universitario de Santiago de Compostela, Santiago de Compostela, Spain; 3 National Tuberculosis and Leprosy Control Program (PNLP in Spanish), Ministry of Health and Social Welfare, Malabo, Equatorial Guinea; 4 National Centre of Tropical Medicine, Instituto de Salud Carlos III (ISCIII), Madrid, Spain; 5 The Spanish Tropical Diseases Research Network (RICET in Spanish), Madrid, Spain; Fundació Institut dInvestigació en Ciències de la Salut Germans Trias i Pujol, Universitat Autònoma de Barcelona, SPAIN

## Abstract

Equatorial Guinea has one of the highest burden of tuberculosis (TB) in Africa. Incomplete adherence to TB treatment has been identified as one of the most serious remaining problem in tuberculosis control. The following study is aimed at determining the adherence to anti-tuberculosis treatment in Equatorial Guinea and its determinants, as well as at assessing the knowledge of the people about the disease. In this cross-sectional study, participants were recruited by non-probabilistic consecutive sampling amongst patients who attended the reference units for TB in Bata and Malabo between March and July 2015. Socio-demographic and clinical data were collected. Adherence to treatment and knowledge about TB were assessed by Morisky-Green-Levine and Batalla tests and a questionnaire on adherence related factors specifically prepared for this research. Descriptive statistics were computed to summarize the data and bivariate analyses by adherence profile were performed with χ2 test for categorical data. A total of 98 patients with TB were interviewed. 63.27% of interviewees had good knowledge about TB (Batalla test) while 78.57% of respondents were adherent according to the Morisky-Green-Levine test. A low educational level, lack of family support and lack of medical advice about the disease were significantly associated to lower adherence level. Patients with re-infection (due to relapse or treatment failure) and those who have suffered from drug shortages were also less adherents. The National Programme for TB Control should consider improving the early diagnosis and follow-up of TB cases, as well as the implementation of all components of DOTS (Directly observed Treatment, short-course) strategy all over the country.

## Background

Despite international efforts, tuberculosis (TB) continues to be one of the main public health problems on a world level, particularly in some low-income countries, where people live in overcrowded conditions with a high prevalence of HIV/AIDS infections and rudimentary health care. According to the last World Health Organization (WHO) report, in 2015 around 9.6 million people in the world suffered from tuberculosis and about 1.5 million people worldwide died yearly from TB. Currently, tuberculosis is the second most common cause of death from infectious disease, after HIV [1].

In Equatorial Guinea (EG), as in other Sub-Saharan countries, the infectious diseases still cause the majority (69%) of deaths and represent the highest disease burden [2]. Despite efforts made in recent years, the incidence of TB has continued to rise to reach an incidence of 144 (132–155) cases for every 100,000 inhabitants in 2013. The percentage of multidrug-resistant tuberculosis (MDR-TB) is estimated to be 1.7% (0.9–2.6) of new cases and 13% (11–15) of previously treated cases [3]. With these figures, the incidence of TB in Equatorial Guinea is comparable to that of countries considered by the WHO to have a high burden of TB (165 cases per 100,000 inhabitants per year) [1].

The most effective method to control TB is by early identification and treatment [4]. Low therapeutic compliance increases TB prevalence and causes the appearance of subtypes resistant to regular chemotherapy [1]. In addition, this implies an increase in morbidity and mortality; and in the costs of TB control programs [5]. This is particularly important in a country like EG, which lacks laboratory capacity to identify drug resistant strains and second line therapeutic options.

Non-adherence is a complex, dynamic phenomenon with a wide range of factors impacting on treatment-taking behaviour (such as medical, relating to adverse effects, and also social determinants) [6]. Traditionally interest in studies undertaken has been centered on biomedical areas, but in recent years there has been increased interest in examining factors which explain the phenomenon of non-adherence. Given the high endemic situation of tuberculosis in EG, with a growing rate of incidence over recent years, in the present study we aimed at looking at different variables which could affect the completion of treatment by patients diagnosed with TB. For this, we intended to identify the medical and social components that explain the phenomenon of non-adherence to anti-tuberculosis chemotherapy in EG and the degree of their effect on it. Knowledge of these factors will help to orientate future actions taken within the framework of the National Programme for TB Control with the purpose of containing the heightened rate of therapeutic failure, the increase of the circulation of drug-resistant strains, and therefore improving the overall control of the disease.

## Methods

### Study area and population

The study was carried out during May–July 2015 in Bata and Malabo, the two main cities in Equatorial Guinea. EG is one of the smallest countries in Africa, with an estimated population of over 1.2 million. It is located in the Gulf of Guinea, in Sub Saharan Africa. Bata is the largest city in the country and it is placed in the Littoral province, while Malabo, the capital, is placed on Bioko Island, in the Bay of Guinea, approximately 25 km from the Cameroon coastline. Despite the fact that EG has a high gross domestic product (GDP) per person, it has similar structural problems as its low income neighbors, with a wide range of people living under the poverty line, a high birth rate, a low life expectancy at birth and a low health expenditure, with an unequal distribution among people [7].

The National Tuberculosis and Leprosy Control Program (PNLP in Spanish) is the program under the department of Public Health in the Equatoguinean Ministry of Health and Welfare (MINSABS in Spanish). Its overall functions are to establish country wide quality free diagnosis and treatment services for TB and Leprosy, and to coordinate the implementation of TB and Leprosy control activities. The program was launched in 1986, and count with a National Directorate and two coordinators, one for each region of the country (mainland and island), who are placed in the two TB reference centers (in Bata and Malabo, main cities of the mainland and the island, respectively). There are also several diagnostic and treatment centers around the country, provided at least with a baciloscopist and a controller. Centers are integrated at a provincial level, therefore, a definite circuit for the transfer and counter transfer of patients exists between health centers at different scales of the system.

The diagnostic capacities are limited as much in the reference centers as in the diagnostic and treatment units. Complementary radiological investigations are rarely carried out, since they are not covered by the National Programme and they are not available everywhere. The samples culture is not available either, so the only way to obtain a microbiological diagnosis is using the Ziehl-Neelsen staining to observe the resistant acid-alcohol bacillus in sputum samples.

### Study design and sample population

We carried out a cross–sectional study aimed at assessing TB therapy adherence in patients attending Bata and Malabo reference units. Participants were recruited by using a non-probabilistic consecutive sampling method as long as they met the following inclusion criteria: having a confirmed diagnosis of TB; being cared for in the TB health care facility in Bata or Malabo; being ≥ 18 years old; and accepting to participate in this study.

### Data collection

During March-July 2015, the patients’ recruitment was performed by the TB technicians while completing routine consultation in the TB reference units. Patients who accepted to participate in the study were informed about the nature and characteristics of the study, and provided their written informed consent. Participation was offered to 98 patients; all of them accepted to be part of this study.

Two trained pollsters, in Bata and Malabo, collected the data individually from each patient. It was obtained through a questionnaire administered to every study participant. This questionnaire was pre-tested on Equatoguinean patients attending to the same health facilities, but not included in this study, for clarity and cultural acceptability. It was composed of three sections. The first form was subdivided into TB patients’ identification information and socio-demographic data. The second part include the Batalla test to measure the patients´ knowledge of tuberculosis, the Morisky-Green-Levine test to assess their attitude towards treatment, and a questionnaire on adherence related factors specifically prepared for this research. Both tests and the questionnaire were previously tested and translated into the main local language, Fang. All patients were given the option to be interviewed in Fang or Spanish, the two official languages of the country.

The Batalla test was originally developed to verify the knowledge of patients about blood pressure and later started to be used as a predictor of adherence and knowledge of individuals about other diseases [8] [9]. The test consists of three questions: Is tuberculosis a lifelong disease? Can tuberculosis be controlled through medication? Could you name one or more organs that may be damaged by tuberculosis? These three questions reflect a patient’s knowledge on TB, and knowledge has been shown to be a predictor of adherence to TB treatment [6] [10]. If the patient correctly answers all questions, she/he is classified as having adequate TB knowledge.

The test of Morisky-Green-Levine is a 4-item test that assess patients´ attitude towards treatment. The Spanish version has been previously validated by Val-Jiménez, et al. [11]. This instrument has been shown to be effective in the detection of non-adherent patients in chronic pathologies such as diabetes and hypertension [12]. The four questions have dichotomous answer, that is, subjects answer either yes or no: Have you ever forgotten to take your medicine for TB? Are you careless about the schedules? When you feel better, do you sometimes stop taking your medicine? Sometimes if you feel worse, do you stop taking your medication? Subjects who answer ‘no’ to all the items are considered as being adherent to the TB therapy.

To identify adherence determinants for TB therapy, a closed ended structured questionnaire was developed after an intensive literature review on the subject. Possible determinants were classified as: related to treatment, to the disease, to the health care and patients´ personnel determinants. All questions had dichotomous answer (yes/no).

Finally, clinical data and information regarding antituberculostatics taken was recorded by the interviewers directly from the patient´s medical record

### Statistical analysis

The collected data were double entered into a data entry file using EpiData software, V.3.1. The data were then transferred to SPSS version 18.0 (SPSS Inc., Chicago, Illinois, USA). Frequencies, means and standard deviations (SD) were computed to summarize the data. Bivariate analyses by adherence profile and level of TB knowledge were performed with χ2 test for categorical data. Where a cell value was below 5, Fisher’s exact test for two–way tables was applied. The criterion for significance was set at p<0.05 based on a two-sided test.

### Ethical clearance

The study was approved by the National Review Board of the Ministry of Health and Social Welfare of Equatorial Guinea. Support letters were obtained from the MINSABS and the National Programme for TB control. Permissions were previously obtained from participating institutions. The researchers worked in collaboration with the clinicians in the identification of TB patients who were on TB therapy. Written informed consent was obtained from all patients prior to study inclusion. The questionnaires were delinked from participants’ identities. Individual responses were not shared with the healthcare workers (HCWs). A summary with aggregated data was shared with the public health authorities and the HCWs after the field work was completed. A written statement was also included on the introductory part of the questionnaires in which further information concerning the purpose of the study and the confidentiality of the research information was given. Data were analyzed in anonymous form.

## Results

### Characteristics of study population

A total of 98 patients with TB, who attended the Tuberculosis Reference Units for medical attention and drug therapy during the months of March-April and June-July at the Malabo and the Bata Hospitals, respectively, were interviewed.

51.20% (n = 50) of the interviewees were women, out of which 37.50% (n = 29) lived on the mainland and 69.05% (n = 21) on the insular zone (p = 0.002). The mean age was 34.35 years (median 31, SD: 12.93). 93.88% of respondents were literate (n = 92). 60.39% and 33.67% reached the secondary level and university level, respectively, being more common a higher study degree in men than in women (p = 0.042). 45.83% of men and 74% of women were not working at the time of the interview (p = 0.004). Other socio-demographic characteristics are summarized in Table 1.

**Table 1:** Main features of the study population, Equatorial Guinea, May-July 2015.

Variables		Total (n = 98)		Sex				p value
				Male (n = 48)		Female (n = 50)		
		n	%	n	%	n	%	
**Sex**	Male	48	48.98	−	−	−	−	−
	Female	50	51.02	−	−	−	−	
**Age**	< = 25 years old	34	34.69	12	25.00	22	44.00	0.137
	26–39 years old	31	31.63	18	37.50	13	26.00	
	> = 40 years old	33	33.67	18	37.50	15	30.00	
**Ethnics**	Fang	64	65.98	31	65.96	33	66.00	0.385
	Bubi	18	17.53	7	12.77	11	22.00	
	Other	16	16.49	10	21.28	6	12.00	
**Religion**	Catholic	74	75.51	37	77.08	37	74.00	0.836
	Evangelist	14	14.29	7	14.58	7	14.00	
	Other	10	10.20	4	8.33	6	12.00	
**Marital status**	Married	29	29.59	18	37.50	11	22.00	0.166
	Single	68	69.39	30	62.50	38	76.00	
	Other	1	1.02	0	0.00	1	2.00	
**Literacy**	No	6	6.12	2	4.17	4	8.00	0.429
	Yes	92	93.88	46	95.83	46	92.00	
**Educational level**	None education	6	6.12	2	4.17	4	8.00	0.042
	Almost Secondary school	59	60.20	24	50.00	35	70.00	
	Almost University degree	33	33.67	22	45.83	11	22.00	
**Currently working**	No	59	60.20	22	45.83	37	74.00	0.004
	Yes	39	39.80	26	54.17	13	26.00	
**Employment status**	Self-employed	15	15.31	8	16.67	7	14.00	0.223
	Worker	19	19.39	13	27.08	6	12.00	
	Unemployed	31	31.63	14	29.17	17	34.00	
	Other	33	33.67	13	27.08	20	40.00	

Regarding the clinical characteristics of the surveyed patients, 83.16% (n = 79), were unaware of the way they had been infected. Women were more likely to point out the contact with a TB patient as a possible transmission pathway than men (p = 0.040). 40.82% (n = 40) reported having a family member who were suffering or had suffered TB. Contact tracing was performed for 46.94% (n = 46) of the cases. Around 13% referred having a TB re-infection (relapse or treatment failure) and 22.45% had co-infection with HIV, while a 15.31% did not know their HIV status at the time of the interview (Table 2).

**Table 2:** TB related clinical characteristics of the study population, Equatorial Guinea, May-July 2015.

Variables		Total (n = 98)		Sex				p value
				Male (n = 48)		Female (n = 50)		
		n	%	n	%	n	%	
Clinical characteristics								
**Possible transmission way**	Contact with TB patient	16	16.84	4	8.70	12	24.49	0.040
	Unknown	79	83.16	42	91.30	37	75.51	
**Any family member with TB**	No	58	59.18	28	58.33	30	60.00	0.867
	Yes	40	40.82	20	41.67	20	40.00	
**Contact tracing**	No	45	45.92	27	56.25	18	36.00	0.058
	Yes	46	46.94	19	39.58	27	54.00	
	Living alone	4	4.08	0	0.00	4	8.00	
	Don´t know	3	3.06	2	4.17	1	4.00	
Re-infection*	No	85	86.73	44	91.67	41	82.00	0.158
	Yes	13	13.27	4	8.33	9	18.00	
**HIV co-infection**	No	61	62.24	30	62.50	31	62.00	0.194
	Yes	22	22.45	8	16.67	14	28.00	
	Don´t know	15	15.31	10	20.83	5	10.00	

* Relapse or treatment failure (it was not distinguished within the patients’ clinical history)

### Knowledge about TB, adherence to TB treatment and associated factors

63.27% of interviewees had good knowledge about TB (Batalla test) while 78.57% of respondents were adherent according to the Morisky-Green-Levine test. For the Batalla test, the worst scored question was the one concerning the identification of the affected organs, while in the case of Morisky test, it was forgetting to take the TB drugs.

The determinants of TB treatment adherence were analyzed and it was found that 91.84% (n = 90) of patients stated that the TB treatment was covered by the Health System, while 14.29% (n = 14) referred to have discontinued treatment because of drug shortages. 40.82% pointed that they knew about the TB treatment side effects and 32.65% (n = 32) said that they had experienced some form of drug-related discomfort.

Around 86% of respondents reported having received treatment recommendations from the doctor. Up to 95% felt motivated to comply with the TB treatment, while 62.24% referred to feel depressed for having this disease (Table 3).

**Table 3:** TB knowledge and adherence profile and related determinants, Equatorial Guinea, May-July 2015.

Variables	Total (n = 98)		
	n (yes)		%
**Batalla test**			
TB is a lifelong disease	3	3.06	
Tuberculosis can be controlled through medication	96	97.96	
Could you name one or more organs that may be damaged by TB?	62	63.27	
Good knowledge on TB (according to Batalla test)	62	63.27	
**Morisky-Green-Levine test**			
Do you ever forget to take the TB drugs?	15	15.31	
Are you careless about the schedules?	12	12.24	
If you feel good, do you stop taking the TB drugs?	1	1.02	
If you feel bad, do you stop taking the TB drugs?	2	2.04	
Adherent (Morisky-Green-Levine test)	77	78.57	
**Determinants of TB therapy compliance**			
**A. Related to the treatment**			
Do you consider that the amount of TB drugs you´re taking is too much?	49	50.00	
Are TB drugs covered by your health insurance?	90	91.84	
Have you ever paid for the TB drugs?	98	100.00	
Are you aware of any TB therapy side-effects?	40	40.82	
Have you ever stop taking TB drugs due to drugs´ shortage?	14	14.29	
Have you ever feel any discomfort after taking your TB drugs?	32	32.65	
**B. Related to the disease**			
Do you cough often regardless the TB treatment?	23	23.47	
Have you failed in previous TB treatment?	6	6.12	
**C. Related to the health care facility**			
Have you ever received treatment recommendations by your doctor?	85	86.73	
Do you think that the doctor is receptive to your questions and concerns?	95	96.94	
**D. Related to the patient**			
Do you feel motivated to comply with treatment?	94	95.92	
Do you feel depressed for having the disease?	61	62.24	
Do you think that this disease has limited your daily activities?	71	72.45	
Have you noticed changes in your lifestyle due to this illness?	69	70.41	
Do you know the diet to comply with the treatment?	69	70.41	
Do you feel emotionally supported by your family	85	86.73	
Is the TB reference unit far from your home?	52	53.06	

In the bivariate analysis, we found a significant association between illiteracy and inadequate TB knowledge according to the Batalla test; patients that could not read or write were more likely to have worst knowledge about TB than patients who were literate (p = 0.001). Moreover, 100% of patients who had no educational degree and 37.39% of those with at least a secondary level education had inadequate TB knowledge compared to 24.24% of those with a university degree (p = 0.002). Regarding Morisky test, no significant differences were observed in the level of adherence by socio-demographic variables. Concerning the clinical characteristics, according to this test, 46.15% of the re-infected cases were found to be non-adherent compared to 17.65% of those patients with no re-infection (p = 0.020) (Table 4).

**Table 4:** Socio-demographic and clinical factors related to TB knowledge and treatment adherence, Equatorial Guinea, May-July 2015.

Variables		Inadequate TB knowledge (Batalla)			Non-adherent (Morisky)		
		n	%	p value	n	%	p value
**Socio-demographic characteristics**							
**Sex**	Male	16	33.33	0.494	12	25	0.399
	Female	20	40		9	18	
**Age**	< = 25 years old	8	23.53	0.136	8	23.53	0.545
	26–39 years old	13	41.94		8	25.81	
	> = 40 years old	15	45.45		5	15.15	
**Zone**	Insular	15	35.71	0.856	9	21.43	0.963
	Continental	21	37.50		12	21.82	
**Ethnics**	Fang	25	39.06	0.567	12	18.75	0.550
	Bubi	7	38.89		4	22.22	
	Other	4	25.00		5	31.25	
**Religion**	Catholic	28	37.84	0.480	17	22.97	0.644
	Evangelist	6	42.86		3	21.43	
	Other	2	20.00		1	10.00	
**Marital status**	Married	11	37.93	0.406	7	24.14	0.807
	Single	24	35.29		14	20.59	
	Other	1	100.00		0	0	
**Literacy**	No	6	100.00	0.001	1	16.67	0.769
	Yes	30	32.61		20	21.74	
**Educational level**	None education	6	100.00	0.002	1	16.67	0.312
	Almost Secondary school	22	37.29		10	16.95	
	Almost University degree	8	24.24		10	30.30	
**Currently working**	No	22	37.29	0.889	13	22.03	0.857
	Yes	14	35.90		8	20.51	
**Employment status**	Self-employed	3	20.00	0.519	2	13.33	0.319
	Worker	7	36.84		4	21.05	
	Unemployed	13	41.94		10	32.26	
	Other	13	39.39		5	15.15	
**Clinical characteristics**							
Re-infection*	No	29	34.12	0.169	15	17.65	0.020
	Yes	7	53.85		6	46.15	
**HIV coinfection**	No	23	37.70	0.951	12	19.67	0.137
	Yes	8	36.36		3	13.64	
	Don´t know	5	33.33		6	40.00	

* Relapse or treatment failure (it was not distinguished within the patients’ clinical history)

Table 5 shows the relationship between the knowledge about TB and the degree of TB adherence (measured with Batalla and Morisky tests, respectively) and several factors related to TB drugs compliance. According to the Batalla test results, it was observed that 22.22% of the patients with inadequate TB knowledge did not receive recommendations from the healthcare professional compared to 8.06% of those who referred an adequate TB knowledge (p = 0.046).

**Table 5:** TB knowledge and adherence determinants, Equatorial Guinea, May-July 2015.

TB treatment adherence related factors		Batalla test					Morisky-Green-Levine test				
		Non-adherent		Aadequate TB knowledge		p value	Non-adherent		Adherent		p value
		n	%	n	%		n	%	n	%	
**A. Related to the treatment**											
Are TB drugs covered by your health insurance?	No	2	5.56	6	9.68	0.472	1	4.76	7	9.09	0.521
	Yes	34	94.44	56	90.32		20	95.24	70	90.91	
Are you aware of any TB therapy side-effects?	No	25	69.44	33	53.23	0.115	14	66.67	44	57.14	0.431
	Yes	11	30.56	29	46.77		7	33.33	33	42.86	
Have you ever stop taking TB drugs due to drugs’ shortage?	No	31	86.11	53	85.48	0.932	15	71.43	69	89.61	0.035**
	Yes	5	13.89	9	14.52		6	28.57	8	10.39	
Have you ever feel any discomfort after taking your TB drugs?	No	23	63.89	43	69.35	0.578	14	66.67	52	67.53	0.940
	Yes	13	36.11	19	30.65		7	33.33	25	32.47	
Have you failed in previous TB treatment?	No	34	94.44	58	93.55	0.858	18	85.71	74	96.10	0.078*
	Yes	2	5.56	4	6.45		3	14.29	3	3.90	
Do you know the diet to comply with the treatment?	No	13	36.11	16	25.81	0.281	11	52.38	18	23.38	0.010**
	Yes	23	63.89	46	74.19		10	47.62	59	76.62	
**B. Related to the health care facility**											
Have you ever received treatment recommendations by your doctor?	No	8	22.22	5	8.06	0.046**	5	23.81	8	10.39	0.100*
	Yes	28	77.78	57	91.94		16	76.19	69	89.61	
Do you think that the doctor is receptive to your questions and concerns?	No	1	2.78	2	3.23	0.901	0	0.00	3	3.90	0.358
	Yes	35	97.22	60	96.77		21	100.00	74	96.10	
**C. Related to the patient**											
Do you feel motivated to comply with treatment?	No	2	5.56	2	3.23	0.574	3	14.29	1	1.30	0.008**
	Yes	34	94.44	60	96.77		18	85.71	76	98.70	
Do you feel depressed for having the disease?	No	13	36.11	24	38.71	0.798	11	52.38	26	33.77	0.119
	Yes	23	63.89	38	61.29		10	47.62	51	66.23	
Have you noticed changes in your lifestyle due to this illness?	No	11	30.56	18	29.03	0.873	10	47.62	19	24.68	0.041**
	Yes	25	69.44	44	70.97		11	52.38	58	75.32	
Do you feel emotionally supported by your family?	No	7	19.44	6	9.68	0.169	6	28.57	7	9.09	0.020**

* p<0.10

** p<0.05

Regarding the Morisky test, the patients who had to discontinue treatment due to drug shortages, and those who didn’t know the diet to be followed were non-adherent more frequently than those who did not suffer such drugs’ shortage (p = 0.035) and those who knew the diet (p = 0.010). In addition, according to this test, among non-adherent patients, it was more common not to have been motivated to comply with treatment (p = 0.008), not having changed their lifestyle (p = 0.041) and not having family support (p = 0.020) than in the adherent patients. Among the adherent patients, 89.61% referred that they had ever received information about the treatment, compared to 76.19% of the non-adherent patients, although this difference was not significant (p = 0.100).

## Discussion

Around one third of the interviewed patients were non-adherent to TB treatment and/or had inadequate TB knowledge. Some individual characteristics, such as the educational level, seem to influence their TB knowledge and level of adherence. Several factors related to health care, with treatment and the patient also showed a significant association with TB knowledge and adherence to anti-TB therapy. These associations have been previously described in other Sub-Saharan countries [13]—[14], but to our knowledge, this is the first study carried out in Equatorial Guinea on this topic.

We found no differences in adherence to anti-TB therapy between men and women, neither in the distribution of TB cases. The majority of the participating women told us they were not working at the time of the interview (p = 0, 004); this data is consistent with the national survey of health in 2011 [15]. This may be due to the disease itself or to gender issues. In terms of age, three-fourths of the cases occurred among adults under age 40. In Equatorial Guinea, the life expectancy at birth is 55 years for men and 57 for women [16], so the population is overwhelmingly young. The highest incidence of TB in young people is typical of developing countries [17]. On the other hand, we have not found significant differences in the treatment adherence by age groups, in agreement with some previous studies in Sub-Saharan area [18], although an increase of the risk of default with increasing age has also been described in Nigeria [19].

22.45% of respondents suffered from co-infection with HIV, although this percentage could be higher, as 15.45% did not know their serological status. This data doubles the co-infection rate registered by Tudo and collaborators in 1999 in the country [20]. Given the high prevalence of infection by HIV in EG, with 6.2% of the population in reproductive age infected [21], it would be desirable to introduce screening for latent tuberculosis infection in HIV patients, ensuring proper adherence to antiretroviral therapy, which in EG is available free of charge [21]. In addition, proper follow up of HIV infection can cause a greater compliance, as describe in a retrospective cohort study based in Yaoundé (Cameroon) [14].

We found that most TB patients lack important knowledge about the disease and its treatment; about 83.16% of patients unknown the transmission path, and the question concerning the identification of the affected organs was the worst scored one according to the Batalla test. This association has also been described in a similar study in Ethiopia [22]. This occurs despite the fact that many of the patients spent the first phase of treatment hospitalized into specific reference centers. Hasket et al. suggest in a study carried out in Uzbekistan that this lack of knowledge can be due to poor communication between health care staff and patients, or to the fact that the information received is partially incorrect or inappropriate [23]. In our study most patients referred that the doctor was receptive to their concerns, and also to have received treatment recommendations, although this was significantly more common among adherent patients. Similar results were also observed in Ghana [24]. Thus, it seems that the given information is important but not enough if health care professionals don’t make sure that the provided message is simple and adapted to the patients understanding.

We found that a lower educational level was significantly associated with lower TB knowledge and treatment adherence. Feeling motivated to comply with treatment and emotional support were also associated with better level of treatment adherence. Family support, including financial assistance, self-motivation, and emotional support, have been previously described to have a strong influence on patient adherence to TB treatment in low-income countries [25–26]. There are also several recent works speaking about the importance of empowering the patient to make their own choices, on the contrary to the traditional organization of many units of TB, focused on protection and compliance with common therapeutic programmes, that often are not adapted to the individual characteristics [6–27]. To fix this, it would be appropriate to implement information and awareness campaigns through the media mass or teaching workshops in the villages’ health posts, for example. In addition, continuous training to community health workers together with social workers should be provided. This strategy is similar to other programs, like those launched to fight against HIV/AIDS or malaria [28]. But ultimately the socio-medical aspects are which should put the focus, since the most important determinants of adherence cannot be corrected through individual interventions on the patient [29, 30].

In addition to sociodemographic variables, we find a greater proportion of patients defaulting among those who were suffering a second episode of tuberculosis. This may be due to the presence of drug-resistant forms [31], which causes a torpid evolution of disease that discourages the patient to continue with a treatment that does not improve their health status. It would be necessary to adapt the treatment of these patients with a second drug, treating adverse effects as well as related co-morbidities, like suggested by Meressa D et al. in Ethiopia [32]. Unfortunately, second-line anti tuberculosis medication is not available for the moment in EG. As expected, we also found a greater percentage of non-adherence among patients who had to necessarily leave the treatment during the drug shortages. Difficulties obtaining drugs to treat both drug-susceptible and drug-resistant disease are major risks in this type of contexts [33]. A sustainable solution that will maintain an uninterrupted supply of anti-TB drugs should be a priority for health policy makers and planners. On the other hand, contact tracing was performed in less than half our study population, and most patients referred unknown transmission way. According to the WHO, more effective combating tuberculosis mechanisms are the identification and early and proper treatment of cases [34]. Thus, strengthened TB surveillance, including data on drug resistance, is also highly recommended in EG.

### Limitations

One of the main limitation of our study is the selection bias, as the patients were recruited from the clinic in the TB unit, thus we may expect that all patients who voluntarily decided to attend were also the most adherent. Another major limitation of this study is measuring adherence with an indirect method such as a personal survey, and that adherence was not actually recorded in any manner via a visual analog scale, or self-reported adherence scores (e.g., over the past 7 or 30 days). Thus, adherence was not verified or validated in any objective way aside from the Morisky scale. Unfortunately, there is no method of reference and indirect methods are the most highly recommended in literature [35]. Additionally, the cross-sectional nature of this data does not allow us to examine causality in the relationship between low adherence and diverse risk factors. Therefore, further research is desirable.

### Conclusions and recommendations

Rates of non-adherence to TB treatment in Equatorial Guinea are above values considered by the WHO as unacceptable [1]. Adherence to TB therapy is a complex and dynamic phenomenon. In general, we can say that it is a chain of responsibility that includes the behavior of the patient, the attitude of HCWs, health policy and a good number of social determinants. Political action must drive the change that overcomes all the problems which we have mentioned. The therapeutic abandonment should be deemed a failure of the system because the system is that leaves the patient, and not so much the patient who deliberately decides leave the treatment [1]. TB is a curable disease and its incidence can be reduced significantly. In addition, it is important to remember that according to the World Bank, the fight against TB is one of the most efficient public health interventions; it costs less than US$1 per day of healthy life gained [36].

The DOTS (Directly observed Treatment, short-course) strategy is another well-known pillars of the fight against tuberculosis, which currently is only ensured in EG in patients admitted in the hospitals during the first phase of treatment. Moreover, DOTS strategy must be accompanied by improvements in social determinants described, as by alone is not enough to ensure an increase in the adherence rates [37]. All cases of re-treatment should be also followed up exhaustively, hospitalizing them if necessary with appropriate respiratory isolation, with the objective of containing this serious problem which is being posed by multidrug-resistant strains in EG.

Any strategy to improve adherence needs to improve health services and ensure that services are appropriate to patients’ needs. This study could also guide the authorities what groups must be directed this strategy, among which meet more risk factors for non-adherence; i.e., persons of low educational level, second time treated cases or people at high risk of social marginalization or little family support. Likewise, several more flexible treatment plans -short and simplified- adapted to the characteristics of each patient should be established.
